# Advance care planning in people with advanced cancer: a phenomenological study

**DOI:** 10.15649/cuidarte.4455

**Published:** 2025-09-01

**Authors:** Mauricio Arias-Rojas, Sonia Carreño-Moreno, Edith Arredondo Holguín

**Affiliations:** 1 Universidad de Antioquia, Medellín, Colombia. E-mail: emauricio.arias@udea.edu.co Universidad de Antioquia Medellín Colombia emauricio.arias@udea.edu.co; 2 Universidad Nacional de Colombia, Bogotá, Colombia. E-mail: spcarrenom@unal.edu.co Universidad Nacional de Colombia Bogotá Colombia spcarrenom@unal.edu.co; 3 Universidad de Antioquia, Medellín, Colombia. E-mail: edith.arredondo@udea.edu.co Universidad de Antioquia Medellín Colombia edith.arredondo@udea.edu.co

**Keywords:** Advance Care Planning, Advance Directives, Palliative Care, Qualitative Research, Planificación Anticipada de Atención, Directivas Anticipadas, Cuidados Paliativos, Investigación Cualitativa, Planejamento Antecipado de Cuidados, Diretivas Antecipadas, Cuidados Paliativos, Pesquisa Qualitativa

## Abstract

**Introduction:**

Significant challenges currently exist in addressing patients' preferences regarding end-of-life care, particularly in the context of advance care planning.

**Objective:**

To understand advance care planning at the end of life in people with cancer in palliative care in Medellin Colombia.

**Materials and Methods:**

This study used a hermeneutic phenomenological approach to explore the lived experiences of nine patients with advanced cancer. In-depth interviews were conducted, focusing on patients' understanding of advanced care planning. Data was analyzed using thematic analysis to identify major themes related to advance care planning.

**Results:**

Six key themes emerged from the data: (1) Clinging to life; (2) Decision delegation and reflective avoidance; (3) Silence to avoid suffering; (4) Resignation; (5) A quiet place; and (6) My legacy. These results describe the experiences of cancer patients in palliative care versus advanced care planning.

**Discussion:**

The findings highlight the complexity of advanced care planning in advanced cancer patients in palliative care.

**Conclusion:**

This study revealed barriers to care planning, including a lack of understanding of the palliative care process, communication difficulties, and cultural factors.

## Resumen

**Introducción:**

Actualmente existen retos significativos para abordar las preferencias de los pacientes con respecto a los cuidados al final de la vida, particularmente en el contexto de la planificación anticipada de cuidados.

**Objetivo:**

Comprender la planificación avanzada de la atención en el final de la vida en personas con cáncer en cuidados paliativos en Medellín Colombia.

**Materiales y métodos:**

Este estudio empleó un enfoque fenomenológico hermenéutico para explorar las experiencias vividas de nueve pacientes con cáncer avanzado. Se realizaron entrevistas a profundidad, centradas en la comprensión de los pacientes sobre la planificación anticipada de los cuidados. Los datos se analizaron mediante análisis temático para identificar los temas principales relacionados con la planificación anticipada de los cuidados.

**Resultados:**

De los datos surgieron seis temas clave: (1) Aferrarse a la vida, (2) Delegación de decisiones y evitación reflexiva; (3) Silencio para evitar el sufrimiento; (4) Resignación; (5) Un lugar tranquilo; y (6) Mi legado. Estos resultados describen las experiencias de los pacientes con cáncer en cuidados paliativos frente a la planificación anticipada de cuidados.

**Discusión:**

Los hallazgos destacan la complejidad de la planificación anticipada de cuidados en pacientes con cáncer avanzado en cuidados paliativos.

**Conclusiones:**

Este estudio reveló barreras para la planificación de la atención, incluyendo una falta de comprensión del proceso de cuidados paliativos, dificultades en la comunicación y factores culturales.

## Resumo

**Introdução:**

Atualmente, existem desafios significativos para abordar as preferências dos pacientes em relação aos cuidados no fim da vida, particularmente no contexto do planejamento avançado de cuidados.

**Objetivo:**

Compreender o planejamento avançado de cuidados no fim da vida em pessoas com câncer em cuidados paliativos em Medellín, Colômbia.

**Materiais e métodos:**

Este estudo utilizou uma abordagem fenomenológica hermenêutica para explorar as experiências vividas por nove pacientes com câncer avançado. Foram realizadas entrevistas em profundidade, com foco na compreensão dos pacientes sobre o planejamento avançado de cuidados. Os dados foram analisados por meio de análise temática para identificar os principais temas relacionados ao planejamento avançado de cuidados.

**Resultados:**

Seis temas principais emergiram dos dados: (1) Apegar-se à vida; (2) Delegar decisões e evitar reflexivamente; (3) Silêncio para evitar o sofrimento; (4) Resignação; (5) Um lugar tranquilo; e (6) Meu legado. Esses resultados descrevem as experiências de pacientes com câncer em cuidados paliativos com o planejamento avançado de cuidados.

**Discussão:**

Os resultados destacam a complexidade do planejamento antecipado de cuidados para pacientes com câncer avançado em cuidados paliativos.

**Conclusões:**

Este estudo revelou barreiras ao planejamento de cuidados, incluindo a falta de compreensão do processo de cuidados paliativos, dificuldades de comunicação e fatores culturais.

## Introduction

The global cancer burden is increasing, and this represents one of the priority issues on the agendas of health decision-makers. According to the World Health Organization (WHO)^1^, cancer is one of the leading causes of death in more than 60% of countries and has displaced cardiovascular diseases, which traditionally held this position. Its growth is a direct effect of socioeconomic development and, with it, population aging, exposure to risk factors, and lack of adherence to healthy behaviors. According to estimates from the Global Cancer Institute^2^ (GCI), cancer is one of the leading causes of death in more than 60% of countries and has displaced cardiovascular diseases, which traditionally held this position. Observatory (GLOBOCAN), 19.3 million new cases and nearly 10 million deaths were recorded in 2020, a situation that is projected to result in a 47% absolute increase in cases by 2040^3^.With the increase in cases, but also with advances in cancer control treatments, the life expectancy of people with cancer is increasing, and palliative care (PC) is more in demand. According to the WHO^4^ In its global atlas of PC, the proportion of people requiring this care annually is 377 adults per 100,000 people over 15 years of age. 

As a result of the growing need for PC, various problems related to this type of care have emerged. The literature highlights various difficulties such as inadequate symptom management, discrepancies between the PC that patients and their families desire and what they receive, noncompliance with preferences regarding the place of death, cases of dysthanasia, lack of counseling, insufficient financial and instrumental support, as well as challenges in decision-making and bereavement support for families^5^. In this context, the erroneous belief that PC is a service exclusive to hospitals limits its access, hinders the meeting of the needs of patients and families, and complicates conversations about concerns and wishes at the end of life. This results in care that is far from the values and goals of those who need this care^6^. Furthermore, PC services face the challenge of improving end-of-life communication, which, despite being recognized as an essential skill for health professionals, continues to encounter multiple barriers to its effective development^7^. In fact, a systematic review found that fewer than 40% of cancer patients have had end-of-life discussions with their physicians^8^. 

Despite the multiple benefits of advance care planning (ACP) for the end of life, its implementation has been a major challenge for high-income countries. This includes difficulties in treatment expectations, understanding of the disease, uncertainty about the prognosis and progression of the disease towards the end of life, lack of knowledge about the optimal time to perform ACP, communication barriers with the healthcare team, and heterogeneity in ACP protocols and programs regarding the objective, approach, and documentation of conversations^9^. In low- and middle-income countries, the problem of the lack of advance care planning for the end of life is exacerbated by limited regulation and research in this area. Furthermore, there are still difficulties in understanding the meaning and scope of ACP from the perspective of patients and healthcare professionals^10^. Middle-income countries such as Colombia have a low level of integration of PCs into the healthcare system^4^, a limited supply of services, limited human resource training, and a limited ability to express and fulfill advance directives. According to the above, for healthcare professionals, recognizing patients' end-of-life experiences with their wishes and desires would improve understanding and advance care planning, addressing communication barriers and facilitating the fulfillment of these wishes. The objective of this study was to understand advanced care planning at the end-of-life among people with cancer in palliative care in Medellín, Colombia. 

## Materials and Methods

A qualitative approach using a hermeneutic phenomenology method was used in this study^11^. To address PAA, it was identified as a multifactorial phenomenon, in which not only the experience of comfort and symptoms converge, but also the frameworks of values, beliefs, laws, and cultures, which validates the need for a subjective approach to its knowledge to understand it. The design was based on the principles of the phenomenological attitude^12^, focused on meanings^13^ as a subjective formation that arises in the consciousness of the person experiencing the experience. 

The hermeneutic phenomenological method was operationalized through the phases of clarifying assumptions about ADP in people with cancer, collecting anecdotes or experiences about their wishes, and conducting a phenomenological interview to gather participants' interpretations of their advance directives^14^. Another phase was the structural phase, understood as a moment of phenomenological reflection on the experience and its understanding based on its meaning, and producing a phenomenological text as a description of the actions, intentions, and experiences of people with cancer in palliative care regarding their advance directives. 

This study was conducted in a specialized cancer care hospital in Medellín, Colombia, for six months and began in January 2023. Cancer patients over 18 years of age, with a palliative prognostic index of less than four points, that is, with a survival prediction greater than 6 weeks^15^, and who were hospitalized in an oncology and palliative care unit due to disease progression, were invited to participate. The study sampling was developed in accordance with that proposed by Moser et al.,^16^ who state that for qualitative research, samples between 6 and 20 participants are usually sufficient. In addition, the saturation criterion was considered^17^, which establishes that data collection should continue until no new ideas, themes, or relevant categories are obtained from the interviews conducted and their analysis. This is frequently a criterion to consider in studies with a qualitative approach to ensure that the sample size has sufficiently captured the complexity of the phenomenon. 

Participants were invited to study through a nurse from the inpatient department, who presented the study objectives and invited the patients to participate. Those patients who agreed to participate in the study were visited by one of the researchers (M.A.R), who reviewed the inclusion criteria, clarified the study objectives, and allowed any questions to be resolved. The participants signed written informed consent. Afterward, the researcher scheduled a repeat meeting with the participant. At this meeting, an in-depth interview^14^ was conducted by the principal investigator (M.A.R.) in the patients' rooms. The interview began with a brief contextualization of the study objectives and began with the question, "Can you tell us about your experience planning your future care during this illness?" The researcher then explored and asked the participant to elaborate on their responses. The interview was recorded with the participants' permission. Initially, four interviews were conducted. After the initial analysis, five new participants were interviewed. 

The principal researcher and a research assistant transcribed the interviews verbatim. The transcripts were then analyzed using Atlas Ti version 7 software by two of the researchers (S.C.M and M.A.R). Data analysis included both macrothematic and microthematic approaches^12^. First, macrothematic reflection was conducted to gain an overview of the text, allowing the researcher to familiarize themselves with its entire content and identify its overall meaning. Microthematic reflection was then conducted, which involved a thorough reading and rereading of the text. This phase sought to break down the text into smaller units of meaning, extracting elements that reflected the participants' experiences and perceptions. This was followed by thematic expression in scientific language, where the extracted ideas were formulated in scientific and academic terms, allowing the emerging themes to be contrasted with previously existing theoretical knowledge. Finally, themes were integrated, where the identified subthemes and themes were grouped into a single central theme, which summarized the essence of the phenomenon studied. 

The rigor criteria^18^ considered were: 1) balanced integration, that is, comprehensiveness between philosophy, researcher, and research topic; 2) openness regarding the focus and alignment with the topic; 3) contextualization within the practical setting; and 4) resonance, related to the richness of the product in the exposition of its meaning. Furthermore, one of the researchers had experience in phenomenological studies (SCM). All findings were discussed within the research group; when disagreements arose in the development of the topics, they were clarified with the help of the third researcher (EAH). 

The study was approved by an ethics committee of the Faculty of Nursing at the University of Antioquia (Record No. CEI-FE 2021-21), and all participants provided written informed consent. All data collected are freely accessible and available for review at Mendeley Data^19^. 

## Results

 Nine patients with advanced cancer, according to their medical history, who were receiving palliative care for their illness were included in this study. The sample size, consisting of nine participants, was defined based on the information saturation criterion. After the seventh interview, no new themes emerged, and accordingly, two additional participants were invited to the study to ensure that no new themes emerged. Interviews lasted an average of 50 to 60 minutes. All participants in this study had advanced cancer. Six women and three men were included. The mean age of the participants was 55.33 years, and their disease duration ranged from three months to three years. Participants were primarily homemakers (66.66%) and had completed high school (55.55%). Religious affiliations varied widely.Table 1 provides information on the participants included in the study.

**Table 1:** Participant demographics (n=9)

Type of cancer	Sex	Age	Education	Occupation	Time since diagnosis (Years)	Religion
Vulva and kidney	Female	58	undergraduate	Retired	2	Jehovah's Witness
Malignant tumor of the peripheral nerve	Male	25	high school	Home	1.5	None
Colon	Male	70	high school	Home	1	Catholic
Cervix	Female	35	high school	Employee	0.25	Catholic
Breast	Female	63	high school	Home	1	Catholic
Pulmonary	Female	72	high school	Home	3	Christian
Prostate	Male	82	elementary school	Home	3	Agnostic
Neuroendocrine tumor	Female	59	undergraduate	Retired	1	Catholic
Stomach	Female	70	elementary school	Home	1	Catholic

The meaning of PAA for people with cancer in palliative care was presented in six thematic units: clinging to life, delegation of decisions and reflective avoidance, silence to avoid suffering, resignation, quiet place, and my legacy. These themes are described below: 

**Clinging to life**

It was evident that difficulties in early detection and communication of the diagnosis and prognosis by healthcare professionals, coupled with patients' limited understanding of medical terminology, prevented them from clearly understanding what it meant to be in palliative care at the end of life, as well as the fact that treatment no longer had a curative purpose. Uncertain about the prognosis, patients expressed narratives of fighting to the end and clinging to life to overcome death. Patients' narratives about fighting to the end were related to being brave, being strong, not giving in, and even resisting until achieving the impossible. This is observed in one patient's account:* "No, I... we've never talked about that, about saying, 'Do this (end-of-life wishes) ... No!' Well, I want to continue with my treatment until the end; so far, I haven't decided to stop. There are moments of anguish, as I told you, but I want to keep fighting, trying, and maybe even trying more extreme things like that." 63-year-old female patient.*

**Delegation of Decisions and Reflexive Avoidance**

This theme reflects both the transfer of decision-making to third parties, such as healthcare personnel, family, or a divine entity, as well as the avoidance of facing the reality of the end of life. In this regard, limitations in decision-making and, consequently, advance care planning were observed. Among the situations presented were decision-making avoidance, transfer of decision-making to healthcare personnel, to God or a supreme being, and to family. Some participants even commented on clinging to the hope of a longer life to avoid thinking about end-of-life decisions. Decision-avoidance was related to the avoidance of reflecting on the real situation of the end of life, partly due to the hospital's medical paternalism, which did not encourage autonomous decision-making. Furthermore, this reflection was influenced by the difficulty of opening to conversations about the end of life among patients, family, and healthcare personnel.

This was reflected in the patients' statements, who stated they had no end-of-life plans and had not even considered such a possibility. Some indicated they preferred to leave decisions related to their care in the hands of healthcare personnel, while others accepted uncertainty as part of God's will or divine design. One patient expressed it this way:* "...we would ask the doctor, the physician, how far things could go and what he would recommend. I always tell the doctors, 'Doctor, you're the one who knows,' tell me what's next [...] You, who have the experience, tell us what we have to do..." A 25-year-old male patient.*

**Keep quiet to avoid suffering**

Various forms of silent pacts were expressed by participants, who described actions such as postponing conversations about cancer, prognosis, and death, softening language with words like "a little dough" instead of "cancer," and avoiding detailed conversations about death and the dying process. This was done to avoid immediate suffering for the patient or their family members, although suffering and uncertainty were constant throughout the progression of the disease. This is how one participant expressed it: *“Not with my mom, because I'm the one who brings up the topic the most, and she's the one who avoids me. I want to raise her awareness, since she'll oversee his (son), and I haven't been able to because she avoids it every time I talk about it. She tells me you're going to live; you're going to fight it. So, it's that constant struggle, like fighting with people who don't accept that I have a terminal illness." A 35-year-old female patient.*

**Resignation**

The lack of knowledge about the life expectancy, coupled with the pacts of silence, limited the participants' time to reflect on life, death, and the dying process. Within these limitations, the participants hinted that they knew the outcome of the process, although in their speeches they resisted admitting it and used expressions that demonstrated resignation to the inevitable. For some participants, resisting death is perceived as an act of courage that lasts if they have the strength and ends what they call "nothing more to do." Resignation means the forced acceptance of the inevitable (death and the dying process) amidst the desire to continue living. The following excerpt details the resigned speech of one of the participants:* "Oh no, as I say, what was done was done. If they can't do more for me and my life, what does it matter? Whatever happens is God's will, you can't change it." 70-year- old female patient. *

**Quiet place**

Tranquility was a recurring theme among the patients interviewed, but beyond a desirable quality at the end of life, it was an attribute assigned to situations, places, and moments. Participants described this tranquil place as one characterized by minimal intrusion into everyday life. For patients, being and living in this tranquil place meant autonomy, independence, enjoyment, happiness, and comfort. Regarding a specific place, although participants stated that their home would be a tranquil place, they clarified that the hospital could be the tranquil place when symptoms could not be managed at home. It was emphasized that, to be in this tranquil place, it should be free of suffering, understood not only by the absence of physical symptoms, but also well-being in the emotional, social, and spiritual dimensions. This is how one participant refers to being free from suffering: *"As I tell my sister, if you see me in a lot of pain, in a hospital, unconscious, and falling into bed, do everything possible so that I don't suffer, or that you suffer, that would be too much suffering to be in bed for a month. I think about, um ... having that strength to say goodbye peacefully, to die peacefully ..." 70-year-old male patient.*

Finally, it was found that patients feel a desire to be with their family and loved ones, including the healthcare workers who cared for them throughout the process, until their final moments. However, being able to spend time with others is a desire mediated by adequate symptom control, what they described as peace of mind, and a genuine desire to spend as much time together as possible. 

**My legacy**

Building and leaving a legacy is a wish expressed not only for the end of life, but for life itself and beyond death. Participants shared ideas about life after death and about legacy after physical death. Participants described legacy as the memory, teaching, or role model they wish to be after death, and the need for these individuals to continue "being" despite physical death. Among the legacies were environmental stewardship, choosing to be cremated, saving other lives through organ donation, being an inspiration to others, and donating belongings. 

In the following story, a participant expresses their desire for how they would like to be remembered: *“I would like to. I have always transmitted a lot of joy to the people I have been with. I was an elementary school teacher for 40 years and my students remember me for the joy and love with which I treated them, so I transmit that to everyone, and I also transmit it to my husband and my son. So, I would like them to remember me a lot and to be very happy. For me, the legacy is that they are happy, because if I transmitted happiness, then people will really be happy.” Female patient, 58 years old.*

## Discussion

This research described the findings regarding end-of-life palliative care (AAP) for a group of people with cancer receiving palliative care in Medellín, Colombia. In this regard, it is important to mention that the development of palliative care (PC) in Colombia is classified as a development level that, in theory, guarantees widespread provision of this type of care within the health system for those who require it^4^; however, in practice, implementation challenges remain. These challenges affect, among other issues, early referral to PC^20^ and with this the possibility of progressively discussing the PAA with the patient. 

Delays in cancer diagnosis and treatment, combined with other factors such as insufficient training of healthcare personnel in communicating bad news, limited coordinated and synchronous interprofessional work, and beliefs related to death as something only under the control of a supreme being or destiny, mean that in many cases there is ignorance of the prognosis and a difficult adaptation to the objectives of palliative treatment^10^. In fact, within the stories it was evident that some of the participants contemplated curative options and when this option was not possible with the available treatments, they clung to the idea of a miracle that would turn the situation around. The literature describes that the resistance to facing the fact that one is undergoing cancer treatment without curative intention is indicative of a poor coping process with the disease^21^. These coping processes generate emotions such as resignation, resistance to reality, defeat, uncertainty, which, added to the lack of psychoemotional interventions, make it difficult to overcome denial and progress towards considering and implementing a PAA^21^. 

Death and the dying process are situations viewed as distant by the study participants. The participants in this study mostly avoided thinking about the future. In this regard, other studies have documented that due to a lack of control over the future, people choose to leave decision- making to family, healthcare providers, or accept the fate imposed by God^9^. 

Faced with the perception that the end of life is a distant event that does not directly involve them as protagonists in the process, the study participants expressed their wishes and desires without delving into the details. Difficulty in becoming involved in an end-of-life process and in making decisions is common in Latin American culture, given the presence of extreme paternalism and familialism, which leads to overprotection in situations of health and illness. This condition limits human autonomy and their capacity and desire to decide, plan, or execute actions to organize the end of life^9^,^22^.

The limited or nonexistent advance planning for the end of life within the healthcare system, within Latin culture, within the training of healthcare professionals^23^, and within patient care policies in PC, means that conversations on the topic occur late^24^. Furthermore, due to this cultural component, the end of life is a complex topic to discuss and develop in depth with people, leading to conversations predominantly centered on the desire to die peacefully, without suffering, with pain control, and in the company, if possible, of loved ones. From the PC philosophy, this is a fragmented or reductionist view of AAPs^24^; however, it is, in fact, the view held by the study participants, from the limited perspective they have been able to shape, amid the disintegration and limited development of cancer control and PC strategies in Colombia^25^. These contextual limitations mean that death from cancer is situated only in the realm of physical suffering and is not seen as a real problem, with a high prevalence and for which urgent actions are required in the normative, practical and cultural spheres^26^. 

According to the findings of this study, some implications for the care of cancer patients in palliative care are recognized. First, it is important for healthcare professionals to be trained to communicate diagnoses and prognoses in a clear and understandable manner. Lack of clarity limits patients' understanding of their condition and the purpose of palliative treatment. Effective communication is key to informed and autonomous decision-making. Second, this study revealed that some participating patients delegate decisions to their family members or physicians due to a lack of support for their own decision-making. This underscores the need to promote ACP, encouraging open dialogue and respecting patients' values. Furthermore, the narratives of struggle, avoidance, and resignation highlight the importance of psychoemotional support in this process. Third, in Latin American culture, paternalism and family overprotection often hinder patient autonomy in decision-making. Because of the above, it is vital that palliative care strategies respect both patient autonomy and the role of the family, balancing participation in decision-making. Finally, given the late detection of cancer in Colombia, it is essential to advance the implementation of existing public policies regarding advance care planning as part of the palliative process. In 2014, palliative care was regulated in Colombia, along with the right to sign advance directives^27^; however, these initiatives currently mostly occur in advanced stages of the disease and not through early conversations after diagnosis. In fact, in a national survey, 54% of healthcare professionals were unaware of this law and the right to sign advance directives^28^. 

This study acknowledges some limitations to its findings. The patients in this study were hospitalized in a health institution specializing in cancer care, so the context of being hospitalized and in an acute health situation may have made advance care planning a secondary issue due to their current health condition. Furthermore, this study could not validate the information with the participants because some of them died before the researchers provided feedback on the results. However, to ensure the rigor of the study, two additional participants were included and interviewed to confirm that no new information emerged beyond that previously analyzed 

## Conclusions

People with cancer in palliative care in Medellín, Colombia, face late diagnoses and those in advanced stages of the disease, which influences their limited opportunity to plan, express, and implement advance care planning. Regarding specific issues related to advance planning, participants expressed desires to die without pain or suffering, to be in the company of family members, and to be as functional as possible. Furthermore, the study found that a lack of understanding of the prognosis, delegating decisions to third parties, and avoiding end-of-life discussions reflect significant resistance to advance planning in this cultural context. 

This study offers a novel approach by identifying the barriers and cultural factors that limit ANP in patients with advanced cancer in PC. These factors underscore the need to rethink the approaches to communication and emotional support provided to patients and their families, ensuring their understanding of the non-curative nature of advanced cancer. It is also necessary to strengthen the comprehensive provision of PC in the country, in addition to developing strategies for social appropriation of knowledge, so that ANP become a topic present in the daily conversations of people with cancer in palliative care and in the general population. 

Finally, the early and effective incorporation of AAP can transform the end-of-life experience, facilitating a more dignified, less painful, and more consistent process with the patient's wishes. Existing health policies must be implemented more effectively to systematically integrate these practices, considering both cultural barriers and opportunities for improvement in communication and comprehensive care. 
